# Assessment of long-term sickness absence: content and face validity of a new questionnaire based on qualitative data from nominal groups

**DOI:** 10.1186/s12874-019-0852-3

**Published:** 2019-11-08

**Authors:** Kaat Goorts, Charlotte Vanovenberghe, Charlotte Lambreghts, Eline Bruneel, Dorina Rusu, Sofie Vandenbroeck, Lode Godderis

**Affiliations:** 1 Katholieke Universiteit Leuven, Centre for Environment and Health, Kapucijnenvoer 35/5, 3000 Leuven, Belgium; 2 Idewe, External Service for Prevention and Protection at Work, Interleuvenlaan 58, 3001 Heverlee, Belgium; 3 Vlaams Patiëntenplatform vzw, groenveldstraat 15, 3001 Heverlee, Belgium; 4 Département des Sciences de la Santé publique, Université de Liège, Médecine du Travail et environnementale, Liège, Belgium; 5 SPMT-ARISTA, External Service for Prevention and Protection at Work, Rue Royale 196, 1000 Brussels, Belgium

**Keywords:** Questionnaire, Content validity, Nominal group, Long-term sickness absence

## Abstract

**Background:**

Increasing rates of long-term sickness absence are a worldwide problem. Belgium is the first country in Europe that aims to screen its entire population of sick leavers (sick leave > 6 weeks) for the risk of long-term sickness absence in order to focus resources on the high-risk group and to provide adequate return-to-work support. Our aim was to investigate content and face validity of a newly designed questionnaire (Quickscan) using item prioritization of patients and professionals in the field of long-term sickness absence. This questionnaire was developed based on a review of the literature and existing instruments (Goorts et al, J Public Health Res 7:1419, 2018).

**Methods:**

Qualitative data were collected using the nominal group technique. The data were gathered exploring factors that influence return-to work restrictions or opportunities.

**Results:**

Participants indicated 20 out of 21 of the questionnaire factors as important reasons that might influence the return-to-work process. Additionally, 16 factors were discussed that were not yet included in the Quickscan but that might provide useful information on return-to-work issues, according to the participants. In the prioritization of items, we found considerable diversity among participants.

**Conclusions:**

Our findings demonstrate the validity of the Quickscan items to ask patients about important return-to-work barriers or opportunities. However, additional factors were identified that may improve the assessment of risk for long-term sickness absence.

## Background

Increasing rates of long-term sickness absence bring high costs for both society and individuals in 28 European countries [1]. Long-term sickness absence is associated with future unemployment, financial difficulties, psychological problems, and social exclusion [2]. Furthermore, there is evidence that remaining active (e.g., in case of low back pain), promotes recovery [2]. Since a timely return to work both reduces expenditure from sickness benefits and prevents long-term work disability, it is important to prevent long-term sickness absences and encourage return to work [2].

Therefore, in Belgium, new legislation has been implemented requiring physicians of sickness fund organisation and insurance companies assess reintegration possibilities within the first 2 months of sickness absence. If the assessment indicates a potential return to the company, the patient is referred to the occupational health physician, who is considered an essential healthcare provider in the reintegration process. However, in Belgium there is a substantial shortage of physicians assessing disability for sickness fund organisations [3].

A questionnaire to identify high-risk profiles for long-term sickness absence among the large group of employees on sick leave may be a useful contribution to support physicians in occupational health and insurance medicine. As a result of high-risk profile identification, resources (e.g. personnel, funding, services) may be provided more efficiently, and the return-to-work process of employees at high risk could start earlier.

Several reviews have identified factors predicting long-term sickness absence based on questionnaires, such as gender, age, level of education, marital status, number of children and private life strains, perceived health, mental and psychosomatic complaints., [4–6]. A limitation of existing questionnaires is their focus on a specific medical condition, despite a consensus among experts that specific medical conditions are not the most important predictor of long-term sickness absence [7]. Another limitation of existing questionnaires is that they are often developed for a specific health care system, which makes application to other systems difficult.

Therefore, based on predictors identified to date and on existing validated questionnaires, in an earlier research project, we developed and validated (factorial validity, concurrence validity) a new questionnaire, called the “Quickscan” (see supplementary files), for early screening for long-term sickness absence risk in Belgium [8]. The Quickscan is a generic questionnaire for use in the entire population of sick- listed individuals after 6 weeks of sick leave [9]. It consists of 61 items loading on 21 factors of long-term sickness absence [10]. Using factor analysis in a previous study, we were able to categorize these factors into four overarching categories: work-related factors, functioning factors, person-related factors and stressful life events [10]. Content and face validity of this new questionnaire have yet to be established.

Content validity is defined by “the degree to which elements of a questionnaire are relevant to a representative of the targeted construct for a particular assessment purpose” [11]. Content validity of instruments is an important basis for construct validity [12]. In addition, content validation has been conceptualized as a qualitative process that is applicable to all elements of a questionnaire. It therefore includes, but is not limited to, careful specification of constructs, review of scaling procedures by content validity judges, by professionals and members of the population [12]. Expert consultation is considered by some to be the most important determinant of content validity [13].

Face validity is established when an individual who is an expert in field reviews the questionnaire and concludes that it measures the characteristic or trait of interest. Face validity is not considered as an active measure of validity. Therefore, in this study it is combined with content validity [14]. We here aim to explore if the content of our questionnaire is valid according to both professionals/experts and patients in long-term sickness absence. We will thus test both content and face validity based on qualitative data from focus groups.

## Methods

### Design and sample

Twenty-four voluntary patients and five professionals from the Flemish and Walloon region participated in the study. Patients were recruited via patients’ organisations which aim to solve common needs and bottlenecks through advocacy at all relevant policy levels and within all relevant health facilities in Flanders (Vlaams Patiëntenplatform), Wallonia & Brussels (Ligue des Usagers des Services de Santé (LUSS)). Additionally, patients were recruited through various calls for participation on social media. The data were collected from November 29th 2017 until February 7th 2018. Dutch- or French-speaking patients who had been on sick leave for at least 6 weeks were eligible for inclusion in the study. The five professionals were selected on the basis of satisfactory experience with long-term sickness absence through patient contacts.

### Procedure

Five focus groups were organized in both national languages: two focus groups in French and three in Dutch. One French focus group was a mixed group with both patients and professionals. In this group, three patients and five professionals were included. We mixed professionals and patients aiming for new insights in sickness absence experienced and discussed from different perspectives. All other groups were patients-only focus groups. There were no differences in process or data generation in this group compared to that in patient-only groups. The patients were asked to join a focus group meeting at one of the four locations in Belgium. The focus group meetings all lasted approximately 2 hours. As recommended in the publication of Vogt et al. (2004), we conducted focus groups in a relatively informal and comfortable setting, with focus group members seated around a circular table to facilitate the participation of all group members [12]. To facilitate coding and analysis of data, all sessions were audiotaped with participants’ consent.

A nominal group technique was used to conduct the focus groups. The nominal group technique is a structured form of a focus group, based on the ideas of the more familiar Delphi method. This consensus technique is commonly used with health professionals for developing clinical practice and setting priorities [13]. The aim is to determine the degree to which experts agree about a particular issue. It is often used when there is lack of scientific evidence, or because of the complexity of the issue [13]. Contrary to the more familiar Delphi method, the nominal group technique consists of face-to-face meetings to facilitate the discussion and consensus forming. The nominal group consensus technique can be used in patients groups as well as in mixed groups with health professionals [13]. In this case, experts were professionals in the field of long-term sickness absence as well as patients. It is widely recognized that patients can reliably and validly express views on many dimensions of care and factors, although they may not be able to evaluate all aspects of the technical side of care [13]

The same structured questioning rounds were conducted in all focus groups.

#### Round I: individual search for important reasons to (not) return to work

Each individually, participants were asked to write five short sentences on separate post-its describing an important reason to (not) return to work after or during the sick leave period. Next, participants had to read and explain each of their post-its (one by one), and were asked to stick them on the whiteboard. The moderator organized the post-its by theme. Each participant could read only one post-it at a time to ensure that all participants had the chance to speak.

#### Round II: discussing the importance of the factors questioned in the questionnaire

Working individually, the participants were asked to read the questionnaire developed by the researchers. Next, they could add more reasons to the whiteboard that also seemed important to them, but had not yet been mentioned in the previous round. These additional reasons were discussed in a plenary session. In addition, participants could discuss whether the reasons from the first round were sufficiently covered in the questionnaire. Furthermore, other remarks or inquiries about the questionnaire were stated at this point.

#### Round III: individual ranking of priority factors

Working individually, participants selected their top five of most important reasons to (not) return to work from the group list in Round I and II and ranked them in order of importance. This was done by writing down the top five reasons and then adding a number (1–5) next to each outcome to signify its ranking. It was decided to rank the top five reasons rather than the whole list of factors. In this respect, Sanderson et al. (2012) argue that ranking a greater number would become difficult and possibly arbitrary [13]. Finally, the ranking was briefly discussed in the group with the participants.

### Ethical considerations

An informed consent form was provided for all participating patients. Ethical approval was obtained from the SMEC (Social Societal Ethical Committee) (G- 2017 08883) at 17/08/2017.

### Data analysis

To perform data analysis of focus group data, we used a tape-based analytic strategy that involved developing an abridged transcript of the whole discussion [12]. Coders (researchers who encoded the data in N vivo) were provided with a list of themes (i.e. the 21 predicting factors for long-term sick leave used in the Quickscan questionnaire) and definitions of these predicting factors. The coders could add and define new themes to the list, if they felt that the themes/reasons discussed in the focus group did not correspond to any of the codes on the list.

There were two coders for each focus group: 1) one of the main researchers of the project and 2) a fellow researcher to assist. The main researcher of the project coded the transcripts first. Only parts where specific reasons (not) to return to work were discussed were coded. Then, the coded files (marked areas) were sent to the fellow researcher without the main researcher’s codes. The second researcher then coded the marked areas independently. Finally, the coders met to discuss any inconsistencies.

The data from the first two rounds were analyzed using an integrated approach to coding as described by Bradley et al. [15] The textual data concerning patients’ opinions about reasons to (not) return to work was coded using a combination of a priori codes that originated from our previous work as described above (Additional file 1: Table S3) and codes that evolved from constant comparative analysis of the data [15].

The data from Round III (scores 1–5) were summed for each outcome selected across all participants. A percentage of the maximum possible score was then calculated for each outcome ((score from Round 3 / 15 × number of participants) × 100). Transcripts of the focus groups were analysed using NVIVO 11 (QSR international, UK).

## Results

### Descriptives

The mean age of all participants was 49 years, but in every focus group, at least one younger person (between 21 and 35 years of age) was included There were slightly more male than female participants (17 vs 12). The majority of participants had been on sick leave for a long time (several years). Most participants (27 out of 29) were not working at the moment of the interview. Eleven different categories from the ICD-10 scale (International Statistical Classification of Diseases and related health problems) were represented in our focus groups. Only four people were following a return-to-work support program at the time of interviewing. All four were trying part-time-work resumption, combined with partial sickness benefits. Table 1 provides an extensive description of the participant’s characteristics.

**Table 1:** Profile of participants F=Female, M = Male, Age = age in years in 2018

Focus group number	Gender	Age	Disease duration (months)	Currently at work? (Y/N)	Reintegration process started? (Y/N)	Diagnosis (ICD-10) OR Professional expertise)
1	F	41	43	Y	Part-time work resumption	XIII
	M	35	47	N	N	VI
	M	60	13	N	N	II, XIII
	M	51	24	N	N	IV
	M	55	/	Y	N	VI, XIII
2	F	48				Patient organisations
	F	32				Patient organisations
	F	56	126	N	N	XIII
	M	26				Patient organisations
	F	61				Kidney diseases
	M	72	120	N	N	I
	M	74				Pension
	F	50	300	N	N	V
3	F	51	60	Y	Part-time work resumption	XIII, VI
	M	48	/	Y	N	VI
	M	29	/	N	N	V
	M	37	/	Y	Part-time work resumption	XI
	M	59	108	N	N	XIX
4	M	35	8	N	N	V
	M	40	11.5	N	N	II
	F	60	2.5	N	N	XIII
	M	51	19	N	N	V
5	F	64	5	N	N	IX
	F	68	/	N	N	XIII
	F	59	/	Y	Part-time work resumption	II
	M	32	/	Y	N	XVIII
	M	52	108	N	N	IV
	M	54	/	N	N	IX
	F	67	/	Y	N	XI

I infectious and parasitic diseases, II Neoplasms, III Diseases of the blood and blood-forming organs and certain disorders involving the immune mechanism, IV Endocrine, nutritional and metabolic diseases, V Mental and behavioural disorders, VI Diseases of the nervous system, VII Diseases of the eye and adnexa, VIII Diseases of the ear and mastoid process, IX Diseases of the circulatory system, X Diseases of the respiratory system, XI Diseases of the digestive system, XII Diseases of the skin and subcutaneous tissue, XIII Diseases of the musculoskeletal system and connective tissue, XIV Diseases of the genitourinary system, XV Pregnancy, childbirth and the puerperium, XVI conditions originating in the perinatal period, XVII Congenital malformations, deformations and chromosomal abnormalities, XVIII Symptoms, signs and abnormal clinical and laboratory findings, not classified elsewhere, XIX Injury, poisoning and other consequences of external causes, XX External causes of morbidity and mortality, XXI Factors influencing health status and contact with health services, XXII Codes for special purposes

### Confirmed factors present in Quickscan questionnaire

Participants discussed 20 out of 21 factors represented in the questionnaire. Only the factor ‘emotional burden’ was not raised. They thus recognized that 20 factors included in the Quickscan are important to either facilitate or prevent them from returning to work. Table 2 presents the codes used to identify the different factors and the number of focus groups in which they were discussed. All codes were assigned to one of the five following categories: work-related factors, stressful life-event factors, factors related to functioning, person-related factors, or environmental factors. Only four of these categories are reported in Additional file 1: Table S3 because the environmental factors category emerged during constant comparative analysis. We here present some quotes for the factors that were not self-explanatory.

**Table 2:** Codebook of questionnaire factors and items, blue: factors from the focus group (16), white: factors from the questionnaire (21)

Work-related factors			
Nr	Factor	Item	# focus groups
1	Autonomy	Are patients able to choose their own tasks, tempo, order in which they perform tasks?	1
2	Learning and development opportunities	Do patients feel they have the opportunity to develop themselves at work, that they are contributing to a useful entity?	5
3	Social support by management	Do patients feel their management has sympathy for their situation?	5
4	Social support by colleagues	Do patients feel their colleagues have sympathy for their situation?	5
5	Physical workload	Do patients perceive their job as physically demanding? (e.g. lifting, …)	3
6	Workload	How do patients perceive the workload? (e.g. time-pressure, number of tasks, …)	3
7	Terms of employment	How satisfied are the patients with their terms of employment (e.g. salary, …)	1
8	Emotional burden	Do patients perceive their jobs as emotionally demanding?	0
9	Turnover intention profession	Have patients been considering changing jobs? /	1
10	Job satisfaction	Do patients feel good at work?	4
11	Work expectations	Do patients think they will have to catch up a lot of work when they return to their job?	2
1	Active support in return-to-work process	Is there any follow-up and support by an assigned person (employer, disability manager, HR, …) to make sure patients are able to take up tasks gradually?	4
2	Employers attitude towards return to work	Do patients feel the employer is willing to adjust the work floor to their needs?	2
3	Mobility	Is it possible for the patient to come to work?	3
4	Adaptive work environment	Are necessary adaptations in working hours, work place adaptations, adapted tasks, possible on the workfloor according to the patient?	5
Stressful life-event factors			
Nr	Factor	Question	# focus groups
12	Stressful life events	Do patients perceive stressful life events in their private life? (e.g. difficulties in the household)	4
5	Practical issues at home	Do patients perceive issues doing practical tasks at home?	3
6	Financial incentives	Do patients have financial incentives to go back to work (e.g. does the benefit not suffice to support their household?)	4
7	Environment and return to work	How does the environment of the patient feel about the patient returning to work?	2
Functioning factors			
Nr	Factor	Question	# focus groups
13	Health perception patient	How do patients perceive their own health in general?	5
14	Psychological distress	Do patients experience psychological distress? (e.g. depressing thoughts, …)	5
15	Pain perception	How do patients perceive their pain?	5
16	Work-health interference perception	Do patients think that returning to work will worsen their condition?	2
17	Return-to-work needs	Do patients think they will be able to return to their previous job, or are adaptations or a job change necessary?	4
18	Return-to-work expectations	Do patients think they will be able to resume their previous job within 4 weeks?	3
19	Recovery expectations	Do patients perceive the treatment as effective for curing their illness?	3
8	Mental Fatigue	Do patients perceive mental fatigue?	4
9	Medication use	Do patients use medication and does this have a negative influence on their functioning?	3
10	Status support process	Do patient have the feeling of being followed up? (e.g. do they have a diagnosis? Have they seen an occupational health physician?)	5
11	Illness recognition	How does the patient think others perceive their illness?	5
12	Willing to return to work	Are patients motivated to return to work?	5
13	Sustainable return to work	Do patients think that sustainable return to work is possible (e.g. in case of chronic illnesses, unpredictability of the illness)	2
14	Illness-life impact	How do patients feel the illness has an impact on their normal functioning?	2
Person-related factors			
Nr	Factor	Question	# focus groups
20	Fear of colleagues’ expectations	Are patients afraid about what colleagues think about their absence?	4
21	Perfectionism	Do patients have perfectionist characteristics?	2
Environmental factors			
Nr	Factor	Question	# focus groups
15	Regulations	Do patients know about the regulations involved in returning to work?	3
16	Communication different stakeholders	Is there, according to the patients, enough communication between the various stakeholders in their return-to-work process (e.g. communication between different physicians)?	2

Six factors were discussed in all five focus groups. Three of these factors were work-related (social support by management, social support by colleagues, and learning and development opportunities) and three were related to functioning (psychological distress, pain perception, and patient’s health perception).

*“The fact that people don’t accept that you have a chronic illness and all the emotions that are related to that.”~ Psychological distress.*

*“You have to feel better before you can return to work” ~ Health perception patient.*

*“How am I feeling?” ~Health perception patient.*

Four factors were mentioned in four focus groups. One was person-related (Fear of colleagues’ expectations), one was stressful life events, one was functioning-related (return-to-work needs), and one was work-related (job satisfaction).

*“The home situation, whether you have a partner, whether you are alone, or whetheryou have a family” ~ Stressful life events.*

*“Support, both at work ánd at home” ~Stressful life events.*

Three groups discussed workload and physical workload (work-related), and return-to-work expectations and recovery expectations (functioning-related).

*“You are at home, and you want to work, but you are afraid to have a relapse … Then you stay at home a little longer to make sure you are ready to resume your work” ~Return-to-work expectations.*

The participants discussed perfectionism (person-related), work expectations (work-related) and work-health interference (functioning-related) in two focus groups.

*“Self-care is for example that you can combine your treatment with your work” ~ Work-health interference.*

*“The idea that you should be fully recovered before you can go back to work is outdated” ~ Work-health interference.*

One focus group discussed three work-related factors: turnover intention profession, terms of employment and autonomy.

*“We live in a society where everything is about performance, so there is a lot of pressure on the individual. You cannot choose yourself to slow down or to perform a little less. There should be an understanding for people who have been on long-term sick leave and who need some time to get back on their feet.” ~Autonomy.*

When being asked about stimulating or preventing factors to return to work, participants also mentioned factors that could not be assigned to the existing categories in the questionnaire. We were able to distinguish 16 new factors.

Table 2 shows the codes given to the factors that had not yet been included in the Quickscan, but were mentioned by the patients in the focus groups. We assigned all codes to five categories: work-related factors, stressful life-event factors, person-related factors, functioning factors and environmental factors. The 16 new codes are marked in blue in Table 2.

Four additional factors were discussed in all five focus groups: three functioning factors (status support process, willingness to return to work, illness recognition) and one work-related factor (adaptive work environment).

*“Maybe you should ask what seems feasible for the patient in the short, middle and long-term, depending on his condition.” ~ Willingness to return to work.*

*“Very often, the employer only cares about production, but for example in a hospital laboratory, it should be possible to find a reasonable solution, adjusted hours, adjusted schedules; that must be possible in such a context.” ~ Adaptive work environment.*

Financial incentives (stressful-life event related), active support in return-to-work (work-related) and mental fatigue (functioning related) were discussed in four focus groups.

*“I think another factor is wage/income. My neighbor was in the hospital, because of cancer, and he had to start working immediately after chemotherapy because otherwise he could not pay for his treatment. He fainted a few times at work in the beginning. For me, that is not what re-integration should be.” ~Financial incentives.*

*“Someone should really follow up that you are not starting with too much workload in the beginning. Making sure you can build up easily. Sort of a gatekeeper.” ~ Active support in RTW process.*

*“It is important that your supervisor and the people you are working with can assess the situation, so they can intervene if necessary”. ~ Active support in RTW process.*

Practical issues at home (stressful-life event related), medication use (functioning-related), regulations (environmental factors), and mobility (work-related) were mentioned in three focus groups.

*“Not being able to combine the household with re-starting work is another important factor to me.” ~ Practical issues at home.*

Two focus groups discussed the illness-life impact and sustainable return-to-work (functioning-related), communication between various stakeholders (environmental factors), environment and return-to-work (stressful-life event related) as well as employers’ attitude towards return-to-work (work-related).

*“Okay, you are chronically ill, but you still have skills. An electrician with heart problems is still an electrician. Or isn’t he? Many people want to come back to work, they are highly motivated. And there is certainly a place for them. Provided that the employer can also give them a place.” ~ Employers’ attitude towards RTW.*

*“Social network, so not just at home, but also broader, so that everyone supports or can support it in some way, so that you want to take these next steps towards work.” ~ Environment and RTW.*

*“There should be clearer communication between all those different actors, so that it would be easier for us. That would also improve things a lot.” ~Communication among stakeholders.*

*“My occupational physician told me he could not go further with my trajectory without the confirmation of the person who had diagnosed me. But there was no coordination between both physicians at all …*” *~Communication among stakeholders.*

Twenty-six factors out of 37 (21 factors from the Quickscan questionnaire and 16 factors from the focus groups) were ranked as the most important factors, indicating the diversity of priorities for participants.

**Fig. 1:**
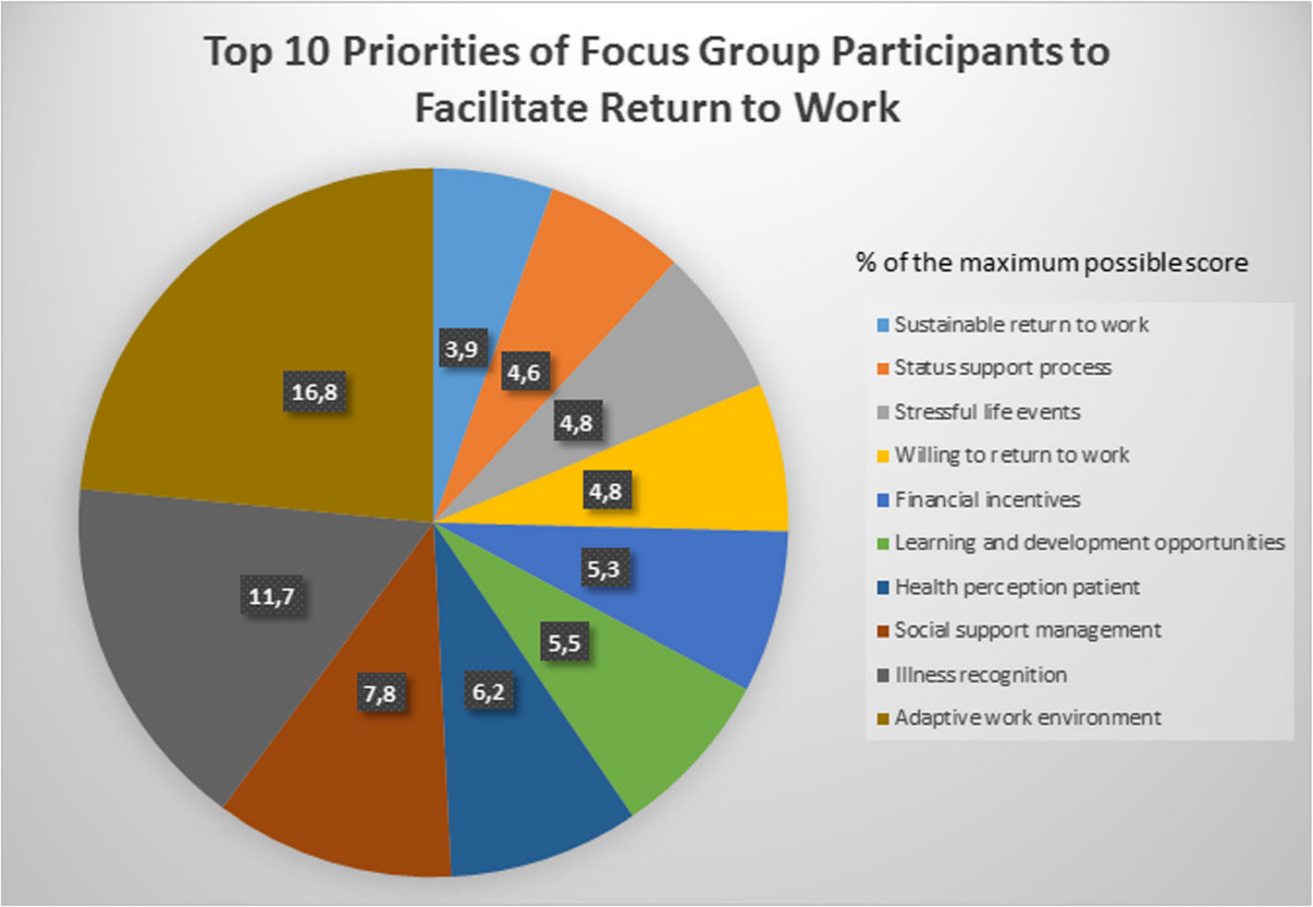
Top 10 priorities of focus group participants to facilitate return to work

Figure 1 shows the top ten priorities. The factors in the top ten represent three out of the five categories: work-related factors (learning and development opportunities, social support management, adaptive work environment), stressful life events (financial incentives, stressful life events), and functioning factors (sustainable return-to-work, status support process, willingness to return to work, health perception patient, illness recognition).

## Discussion

The purpose of this study was to test both content and face validity of the Quickscan questionnaire by asking questions to experts (both patients and professionals) using the nominal group technique. In general, we can conclude that the Quickscan has met the requirements of both face and content validity as described by Haynes et. Al (1995), and Vogt et. Al (2004) [11, 12].

The qualitative data generated from the nominal groups with experts provided insight into the items that patients perceive as influencing their return-to-work process. Although the term ‘expert’ has typically been used to refer to researchers who are knowledgeable in a specific research domain, members of the population under study may also be considered experts in some cases. According to Vogt et al. (2004), some authors have already addressed the importance of consulting members of the population in identification and specification of constructs [12]. According to Haynes et al. (1995), carefully structured open-ended interviews with members of the target population can increase chances that items are content valid for their intended purpose and can also suggest additional facets and the need for construct refinement [11, 12].

### Content validity

First, the items in the Quickscan were found to be valid according to the participants of the focus groups. Hence, 20 out of 21 factors were spontaneously discussed by the participants as important factors that might encourage or prevent them from resuming work. The fact that almost all factors were discussed is a good measure for the questionnaire’s content validity. One factor, emotional burden, (whether the patient perceives work as emotionally demanding) was not discussed as a reason to not resume work. A possible explanation could be that this is only important if the reason for the patient’s sick leave is linked to emotionally demanding work. In addition, only four participants reported that their absence was caused by a mental condition, which might explain why this factor was underexposed. Another possibility is that this factor is not a good predictor for long-term sickness absence.

### Additional items

Participants pointed out 16 new factors during focus group discussion, almost all about the the patient’s perception of the sickness situation (e.g. what kinds of adaptations patients will need to return to work, how they perceive management efforts to realize these adaptations, …). This means that participants consider their perceptions should be questioned rather than taken as objective measures about sick leave.

Earlier studies have shown that the patient’s own estimation about the sick-leave situation is the best predictive factor for long-term sickness absence [16]. This theory seems to be supported by the findings of this qualitative study.

According to Canceliere et al. (2016)., environmental factors associated with positive return-to-work factors included stakeholder participation in the return-to-work process, work modification/accommodation, and return- to-work coordination [17]. These factors can be linked to the additional factors raised in the focus groups: communication among stakeholders, adaptive work environment, and active support in the return-to-work process.

Canceliere et al. (2016) also described the activity limitations/participation restriction (e.g., limited ability to perform activities of daily living (ADLs) and periods of unemployment), and higher physical work demands as an important factor. In our focus groups, patients described practical issues at home, financial incentives and environment and return-to-work [17].

The factors that were mentioned by the patients in this study are thus in line with findings in other studies. It should therefore be considered adding these factors to the questionnaire.

In our focus groups, patients argued that it is important to ask questions about the status of the support process “does the patient feel that they are being followed up?”

Similarly, Canceliere et al. (2016) argue that multidisciplinary interventions involve multiple resources including professionals from more than one discipline (e.g., occupational health physician, case-coordinator, physical therapist and others), who deliver a variety of intervention elements (e.g., exercise, education, behavioral treatment, vocational advice, etc.) with or without the inclusion of other stakeholders (e.g., supervisors, employers, insurance representatives).” [17]. Including a question about the status of the support process might thus be an important issue in the questionnaire.

Another important trend we observed in the additional factors is that although they are not that different from the factors in the questionnaire, patients added more nuance and detail to their description. This might be the reason that the additional factors had not been included in the questionnaire yet. For example, the questionnaire contains an item on the patient’s perception of their relationship with the employer (is he understanding towards the situation of the employee?), but some patients thought that it is also very important how the patient perceives the attitude of the employer towards adjustments on the workplace.

This nuance might make a considerable difference for the patient when he or she thinks the employer will not make an effort to introduce adjustments to the workplace.

Another remarkable factor suggested by patients can be categorized under stressful life events. Patients stated that financial incentives should be questioned as well. In their opinion, a patient who will only resume work out of financial needs will have a high risk of relapse.

Next, we noted two extra factors concerning the patient’s knowledge of regulations governing the return—to-work process. Patients argued that both knowledge about the regulations in return-to-work and communication between the various stakeholders are important factors in the return-to-work process. Hence, if knowledge is not transferred correctly, patients might miss certain opportunities to resume work in adapted circumstances. In addition, improvements in communication may be a success factor for a variety of new interventions [18].

### Patients’ prioritization

From the patients’ prioritization, it has become apparent that there is a large diversity in priorities. Every patient has his or her own story and therefore sees different priorities. This finding stimulates us to include a wide variety of factors in the Quickscan questionnaire.

### Nominal group technique

There were several benefits of using the nominal group technique with patients. First, personal views and priorities could be clarified and group discussions enhanced understanding of others’ perspectives. Second, the patients’ decision-making process was made transparent by recording the group discussion as qualitative data. Third, patients appreciated the invitation to cooperate in the process of developing a new questionnaire. They felt valued in their role as expert concerning their own experiences with sick leave.

The nominal groups enabled a preliminary prioritization of important factors to (not) resume work. Although we will not delete any items in the Quickscan based on the focus group research, it was interesting to test for possible consensus about some factors being more important than others. It is thus essential to determine a consensus level before the priority scores are calculated. In other nominal groups and Delphi studies, consensus levels have ranged from 51 to 75% [13]. Setting a consensus level is an arbitrary decision, but would be aided by the inclusion of researchers’ and patient partners’ opinions. Since the highest percentage in our study is 16.8%, we cannot claim any kind of consensus among participants according to priority setting.

### Strengths and limitations

There were strengths and limitations specific to this study. Two patients dropped out at short notice, one for personal reasons, one because of illness.. This is to be expected during research with people suffering from illness. It is not easy for (chronically) ill people to participate in this kind of research. Most of them are occupied with treatment, diagnosis, and try to manage their personal life. A focus group discussion is very intensive for most of them. We have to take into account that the most severely injured or ill people were not able to participate in our research. Furthermore, we should be aware that most patients had a very long sick leave duration. This was advantageous for the study in that our aim was to analyze factors facilitating or preventing people from returning to work. People in long-term sick leave probably have a lot of experience with different kinds of factors. However, the instrument is intended to be used for patients after 6 weeks of sick leave. It is possible that a group of patients 6 weeks after injury or illness would provide us with different insights.

Including professionals in one of the focus groups might both be a strength and a weakness. On the one hand, it is interesting to include the opinion of people who work with our target audience on a daily basis, but on the other hand, we could not control the possible influence they had on the opinions of patients. However, the results did not seem to differ from those of the focus groups without professionals..

### Future research

Further research should investigate to what extent factors from the focus group (16) should be added to the those of the questionnaire (21). For example, by testing whether adaptive work environments that address one or more of these 16 factors from the focus groups can minimize long-term sickness absence. The outcome of the current research will assist researchers in adding additional factors and improving the instrument.

The patients’ prioritization suggests an interesting hypothesis: “If work-related factors such as an adaptive work environment and support from management address how the patient perceives his/her illness and health, then long-term sickness absence is likely to be minimized.” This theory recognizes that the top reasons for long term sickness absence as reported in Fig. 1 are categorized as work-related and functioning (illness recognition and patient health perception). We believe this hypothesis should be tested using more quantitative approaches.

Our list of 16 additional factors shows that many factors could be related to the self-determination theory (SDT). Deci et al. discussed the SDT relevant to the postulate that all employees have three basic psychological needs—for competence, autonomy, and relatedness—the satisfaction of which promotes autonomous motivation, high-quality performance, and wellness [19]. Mapping this theory on the Quickscan might therefore be a useful exercise. Since the self-determination theory was not used to develop the Quickscan or to code the focus group textual data, the relationship with this theory deserves further research. It would be interesting to analyse if autonomy, relatedness, and competence are maximized for those returning to work if management and colleagues are supportive and if the work-environment is adaptive (i.e. provides opportunities for learning and development).

## Conclusion

In conclusion, the nominal groups not only confirmed the validity of the Quickscan, but also provided the researchers with many new insights on possible additional predicting factors for long-term sickness absence. According to the patients involved in the study, the instrument has considerable potential to function as a screening instrument for long-term sickness absence. Early screening may be key to a better follow-up of patients in need of support to resume work.

## Supplementary information

**Additional file 1: Table S3.** Structure, content, source-questionnaires and scoring* of the questionnaire.

## Data Availability

The datasets generated and/or analysed during the current study are not publicly available because participants might be identifiable through their personal stories, even after anonymization, but are available from the corresponding author on reasonable request.

## Abbreviations

ADL: Activities of Daily Living
ICD-10: International Statistical Classification of Diseases and related health problems (10th revision)
LUSS: Ligue des Usagers des Services de Santé
SDT: self-determination theory
SMEC: Social Societal Ethical Committee
