# Correction to: Assessment of long-term sickness absence: content and face validity of a new questionnaire based on qualitative data from nominal groups

**DOI:** 10.1186/s12874-019-0872-z

**Published:** 2019-12-04

**Authors:** Kaat Goorts, Charlotte Vanovenberghe, Charlotte Lambreghts, Eline Bruneel, Dorina Rusu, Marc Du Bois, Sofie Vandenbroeck, Lode Godderis

**Affiliations:** 1 Katholieke Universiteit Leuven, Centre for Environment and Health, Kapucijnenvoer 35/5, 3000 Leuven, Belgium; 2 Idewe, External Service for Prevention and Protection at Work, Interleuvenlaan 58, 3001 Heverlee, Belgium; 3 Vlaams Patiëntenplatform vzw, groenveldstraat 15, 3001 Heverlee, Belgium; 4 Département des Sciences de la Santé publique, Université de Liège, Médecine du Travail et environnementale, Liège, Belgium; 5 SPMT-ARISTA, External Service for Prevention and Protection at Work, Rue Royale 196, 1000 Brussels, Belgium

**Correction to: BMC Med Res Methodol (2019) 19:205**

**https://doi.org/10.1186/s12874-019-0852-3**

In the original publication of this article [1] the author Marc Du Bois was omitted. In this correction article the author and the corresponding details are provided. The publisher apologizes to the readers and authors for the inconvenience.

The original publication has been corrected.
