# Supplementary material for: Assessment of long-term sickness absence: content and face validity of a new questionnaire based on qualitative data from nominal groups
Source: BMC Med Res Methodol. 2019 Nov 8;19:205. doi: 10.1186/s12874-019-0852-3 (PMC6842172; doi:10.1186/s12874-019-0852-3)
Supplement: Supplementary file 1 — Additional file 1: Table S3. Structure, content, source-questionnaires and scoring* of the questionnaire. [file 12874_2019_852_MOESM1_ESM.docx]

| Work-related factors | Stressful life-events | Functioning | Person-related factors |
| --- | --- | --- | --- |
| Autonomy (5 items)  Absenteeism screening questionnaire  *Is the patient able to choose his own tasks, tempo, order in which he performs his tasks?* | Stressful life-events (8 items) Vragenlijst arbeidsre-integratie [questionnaire re-integration]  *Does the patient perceive stressful life events in his private life? (e.g. difficulties in the household)* | Health perception patient (2 items)  Disability risk questionnaire  *How does the patient perceive his own health in general?* | Fear of colleagues expectations (1 item)  ORO-questionnaire(Obstacles to return to work questionnaire)  *Is the patient afraid about what colleagues think about their absence?* |
| Learning and development opportunities (4 items)  Vragenlijst arbeidsreïntegratie/ vragenlijst beleving en beoordeling van de arbeid [questionnaire re-integration/ questionnaire perception and assessment of labor]  *Does the patient feels as if he has the opportunity to develop himself at work, that he is contributing to a useful entity?* |  | Psychological distress (7 items)  SPOC-NL(Somatic Pre-Occupation and Coping Questionnaire )/brief illness perception questionnaire  *Does the patient experience psychological distress? (e.g. depressing thoughts, …)* | Perfectionism (4 items)  Vragenlijst arbeidsreïntegratie questionnaire re-integration]  *Does the patient have perfectionist characteristics?* |
| Social support management (2 items)  Vragenlijst arbeidsreïntegratie [questionnaire re-integration]  *Does the patient feel as if his management has sympathy for his situation?* |  | Pain Perception (3 items)  SF- 36/ALBPSQ-NL(Acute low back pain screening questionnaire-NL)  *How does the patient perceive his pain?* |  |
| Social support colleagues (2 items)  Vragenlijst arbeidsreïntegratie [questionnaire re-integration]  *Does the patient feel as if his colleagues have sympathy for his situation?* |  | Work-health-interference perception (1 item)  Vragenlijst arbeidsreïntegratie [questionnaire re-integration]  *Does the patient think that returning to work will worsen his condition?* |  |
| Physical workload (7 items)  Vragenlijst beleving en beoordeling van de arbeid [questionnaire perception and assessment of labor]  *Does the patient perceive his job as physically heavy? (e.g. lifting, …)* |  | Return to work needs (1 item)  Vragenlijst arbeidsreïntegratie questionnaire re-integration]  *Does the patient thinks he will be able to return to his previous job, or are adaptation or a job change necessary?* |  |
| Workload (7 items)  Vragenlijst arbeidsreïntegratie/ vragenlijst beleving en beoordeling van de arbeid [questionnaire re-integration/ questionnaire perception and assessment of labor]  *How does the patient perceive the workload at his job? (e.g. time-pressure, number of tasks, …)* |  | Return to work expectations (1 item)  Vragenlijst beleving en beoordeling van de arbeid [questionnaire perception and assessment of labor]  *Does the patient think he will be able to resume his previous job within 4 weeks?* |  |
| Terms of employment (1 items)  Vragenlijst arbeidsreïntegratie [questionnaire re-integration]  *How satisfied is the patient with his terms of employment (e.g. salary, …)* |  | Recovery expectations (1 item)  SPOC-NL(Somatic Pre-Occupation and Coping Questionnaire –NL)  *Does the patient perceives as if his treatment is effective for healing his illness?* |  |
| Emotional burden (1 item)  Vragenlijst beleving en beoordeling van de arbeid [questionnaire perception and assessment of labor]  *Does the patient perceive his job as emotionally demanding?* |  |  |  |
| Turnover intention profession (1 item)  Vragenlijst arbeidsreïntegratie [questionnaire re-integration]  *Has the patient been considering changing jobs?* |  |  |  |
| Job satisfaction (1 item)  Vragenlijst arbeidsreïntegratie [questionnaire re-integration]  *Does the patient feel good at work?* |  |  |  |
| Work expectations (1 item)  ORO-questionnaire(Obstacles to return to work questionnaire)  *Does the patient think he will have to catch up a lot of work when he returns to his previous job?* |  |  |  |
| *All questions are scored on a 6 point Likert scale | | | |

Table 3 Structure, content, source-questionnaires and scoring* of the questionnaire

21 factors divided in four over-arching categories (work-related factors, stressful life-event factors, functioning factors and person-related factors (adapted from different validated questionnaires)
